# Identification of Pesticide Transformation Products in Surface Water Using Suspect Screening Combined with National Monitoring Data

**DOI:** 10.1021/acs.est.1c00466

**Published:** 2021-07-22

**Authors:** Frank Menger, Gustaf Boström, Ove Jonsson, Lutz Ahrens, Karin Wiberg, Jenny Kreuger, Pablo Gago-Ferrero

**Affiliations:** † Department of Aquatic Sciences and Assessment, Swedish University of Agricultural Sciences (SLU), SE-75007 Uppsala, Sweden; ‡ Department of Environmental Chemistry, Institute of Environmental Assessment and Water Research—Severo Ochoa Excellence Center (IDAEA), Spanish Council of Scientific Research (CSIC), Jordi Girona 18−26, 08034 Barcelona, Spain; § Catalan Institute for Water Research (ICRA), Carrer Emili Grahit 101, 17003 Girona, Spain

**Keywords:** nontarget screening, high-resolution mass spectrometry (HRMS), accurate mass, metabolites, TIMFIE, prioritization strategy

## Abstract

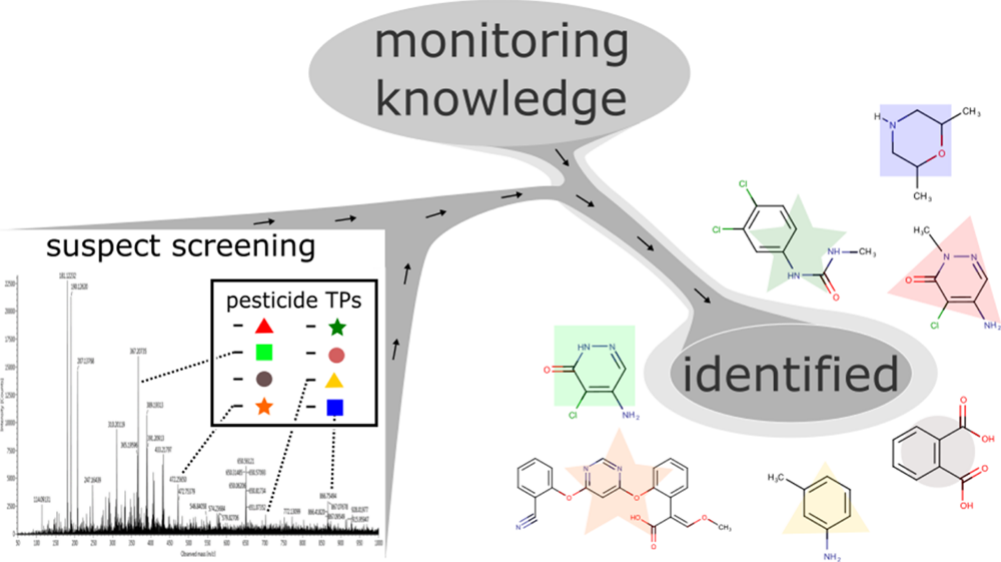

Pesticides are widespread anthropogenic chemicals and well-known environmental contaminants of concern. Much less is known about transformation products (TPs) of pesticides and their presence in the environment. We developed a novel suspect screening approach for not well-explored pesticides (*n* = 16) and pesticide TPs (*n* = 242) by integrating knowledge from national monitoring with high-resolution mass spectrometry data. Weekly time-integrated samples were collected in two Swedish agricultural streams using the novel Time-Integrating, MicroFlow, In-line Extraction (TIMFIE) sampler. The integration of national monitoring data in the screening approach increased the number of prioritized compounds approximately twofold (from 23 to 42). Ultimately, 11 pesticide TPs were confirmed by reference standards and 12 TPs were considered tentatively identified with varying levels of confidence. Semiquantification of the newly confirmed TPs indicated higher concentrations than their corresponding parent pesticides in some cases, which highlights concerns related to (unknown) pesticide TPs in the environment. Some TPs were present in the environment without co-occurrence of their corresponding parent compounds, indicating higher persistency or mobility of the identified TPs. This study showcased the benefits of integrating monitoring knowledge in this type of studies, with advantages for suspect screening performance and the possibility to increase relevance of future monitoring programs.

## Abstract 2

The combination of advanced HRMS-based strategies with monitoring knowledge led to a more comprehensive picture on the pesticide transformation products present in surface water.

## Introduction

A large number of chemical pesticides are regularly used in the European Union (EU) to protect crops and enhance yields, with more than 400 active substances currently approved by the European Commission.^1^ Their demonstrated environmental presence may lead to hazardous effects on nontarget species in different ecosystems, particularly in highly affected aquatic environments.^2,3^ Because of the diverse chemical properties of pesticides, selection of pesticides for environmental monitoring programs poses a challenge. Besides active substances, there is also a vast number of potential transformation products (TPs), whose occurrence and effects in the environment are largely unknown.^4−6^ It is of paramount importance to study these TPs, as they may exceed the concentration levels, persistency, mobility, and toxicity of their parent compounds.^5,7−9^ The identification of pesticide TPs in the environment is a challenging and a nonroutine task. Due to these difficulties and the lack of large data sets in terms of occurrence and effects, some of these substances might not be sufficiently regulated. Thus, there is an urgent need to improve understanding of the presence of pesticide TPs in the environment to improve the validity of risk assessments and to provide valuable information to regulators.

Pesticide-monitoring programs provide detailed knowledge on pesticide occurrence and long-term concentration trends.^10^ This is important for the estimation of ecological risks from the use of pesticides. Although some understanding on the occurrence of pesticide TPs has been reached,^7,11−14^ current monitoring is mainly focused on the active substances of the pesticides, whereas their TPs are largely underrepresented due to lack of knowledge, analytical methods, or resources. The Swedish national monitoring program for pesticides in surface water has been ongoing since 2002 and currently covers about 150 compounds corresponding to most chemicals registered in plant protection products in Sweden (169 registered compounds in 2018) (Boye et al., 2019). Today, only 11 pesticide TPs are well investigated and monitored in the Swedish monitoring program, for example, 2,6-dichlorobenzamide (BAM), deethylatrazine, and deisopropylatrazine.

Target methodologies commonly used in monitoring programs require reference standards, hindering the investigation of most pesticide TPs, for which reference standards are often not available.^15^ Advances in high-resolution mass spectrometry (HRMS) have opened windows of opportunity for the identification of unknown contaminants of emerging concern (e.g., pesticide TPs), and suspect screening has been demonstrated to be a powerful tool to screen for substances suspected to be present in a sample (suspects) without the availability of authentic reference standards.^5,6,15,16^ Although reference standards are not needed a priori, suspect screening has the potential to become more successful if detailed a priori information is considered.^16−18^ Suspect screening using a smartly selected list of suspects can be a powerful screening tool for studies aiming at a specific group of chemicals of interest (e.g., pesticide TPs).^19^

Here, we tested the hypothesis that integrating knowledge and expertise of monitoring programs and regulatory agencies with the evaluation workflow of HRMS-based suspect screening will lead to a more powerful screening for a better understanding of the presence of pesticides and their TPs in the environment. This way of benchmarking monitoring data to improve the success of suspect screening of TPs is a novel approach and was applied in a Swedish context. A suspect list highly relevant for Swedish agricultural areas was built and used in this study to screen time-integrated surface water samples of two Swedish streams. Previously not monitored pesticide TPs were semiquantified after confirmation, and their concentrations were compared to those of their respective parent compounds. Analytical strategies developed in this work allow for improvements of current monitoring programs by increasing the knowledge on relevant compounds to be included in future regular monitoring of pesticides and their TPs.

## Materials and Methods

### Swedish National Monitoring Program for Pesticides

Information about the Swedish national monitoring program for pesticides including detailed information about sampling, extraction, and analysis can be found in the study by Boye et al. (2019). In the year of our study (2017), the accredited methods of the monitoring program covered 142 target substances, and 1-week composite samples were analyzed weekly from May to November (no sampling during August).

### Chemicals and Reference Compounds

Reference standards of target analytes (131 pesticide active substances and 11 TPs) were included based on the monitoring program in 2017 (*n* = 142) and used as references in the suspect screening. Commercially available reference standards for newly, tentatively identified substances (*n* = 26) were purchased and analyzed for final confirmation in the last step of the suspect screening (for details, see Table S1 in Supporting Information-A). Clothianidin-*d*_3_ and imazalil-*d*_5_ obtained from Dr. Ehrenstorfer (Augsburg, Germany) were used in Milli-Q water as internal standard solution (IS) and utilized for semiquantification of newly confirmed compounds. Detailed information on chemicals used during extraction and analysis is included in Supporting Information-A.

### Sampling Locations

Two sampling sites located in two small Swedish streams (i.e., E21 and M42, referred as E and M, respectively) that are part of the Swedish national monitoring program for pesticides were chosen for sampling. Both catchments are located in areas with high agricultural intensity and have been studied extensively as representatives of typical agricultural regions in Sweden since 2002.^10^

The areas of the E and M catchments are 16 and 8.2 km^2^, respectively, and both comprise about 90% agricultural land. Together, the two areas cover a variety of grown crops, with about 60% of the agricultural land in both areas used for cereals and the remaining area mainly used for peas and rapeseed, as well as potatoes (E) and sugar beets (M). Interviews with the farmers in each area are conducted every year, whereby information about, for example, grown crops and pesticide application (date, product, dose, and crop) is collected. The pesticide application is most intensive during spring and early summer, with some application also during summer and autumn (Figure S1 in Supporting Information-A).

### Sampling and Extraction

Samples were collected using the newly developed Time-Integrating, MicroFlow, In-line Extraction (TIMFIE) sampler, previously applied in target analysis of pesticides in water.^20^ Eight weekly samples were collected at each sampling site during two sampling campaigns of four samples each. The first sampling campaign covered the period right after the intensive spring and early summer pesticide applications and was performed during June and July 2017, while the second campaign covered the late summer and autumn pesticide applications and was performed during September and October 2017 (Figure S1 in Supporting Information-A).

The TIMFIE sampler was constructed from a single-use plastic syringe connected to a narrow bore flow restrictor and two solid-phase extraction (SPE) cartridges connected in series. By pulling out the syringe plunger and blocking it with a metal pin, a strong negative pressure was created in the syringe barrel, resulting in a low water flow (typically a few microliters per minute) through the SPE system, and compounds of interest were continuously extracted for 1 week. The two SPE cartridges used in this study were Chromafix HR-P (hydrophobic polystyrene-divinylbenzene copolymer, size small, 50 mg, particle size 50–100 μm) followed by Chromafix HR-XAW (hydrophobic polystyrene-divinylbenzene copolymer with secondary and tertiary ammonium modification, i.e., a weak mixed-mode anion exchange material, WAX, size small, 50 mg, particle size 85 μm), both from Macherey-Nagel (Düren, Germany). The cartridges were conditioned with 5 mL of methanol and 10 mL of ultrapure water prior to use. Approximately, 60 mL of water was sampled and extracted in the field during 1 week. With TIMFIE, the sample volume will differ between samples, but because the extracted water ends up in the syringe, the exact volume for each sample can be determined from the measuring scale on the syringe barrel, thus allowing quantitative analysis. After each sampling week, the TIMFIE samplers were put in zip-lock plastic bags in insulated boxes on ice and sent over night to the analytical laboratory.

In the laboratory, the sampler was disassembled, without separating the two SPE columns, and IS was added to and mixed with the sample water standing in the small void on the inlet side of the first SPE column (HR-P). A 5 mL syringe barrel (PP) was attached to the SPE cartridge, and 5 mL of ultrapure water was then added and slowly pressed through the stacked cartridges to load the ISs and to wash the SPE materials. After centrifugation (5 min at 3000*g*) and drying of SPE adsorbents using nitrogen gas at room temperature, the columns were eluted with 3 mL of methanol/acetone 1:1 (v/v), 2 mL of acetone, and 4 mL of 80 mmol L^–1^ ammonia in methanol. To the pooled extract, 50 μL of dimethyl sulfoxide was added as an evaporation keeper and the extract was evaporated under a gentle stream of nitrogen gas in a water bath set at 40 °C. After adding 100 μL of methanol, the tube was mixed on a vortex, centrifuged briefly, and stored at −20 °C pending instrumental analysis. On the day of analysis, the extracts were diluted with 150 μL of ultrapure water, mixed, centrifuged 3 min at 3000*g*, and transferred to LC vials with 250 μL glass inserts.

### Instrumental Analysis

Instrumental analysis was carried out using a Waters Acquity ultrahigh performance liquid chromatography (UHPLC) system coupled to a Waters Xevo G2-S quadrupole time-of-flight (QTOF) mass spectrometer equipped with an electrospray ionization (ESI) interface and data were acquired in MS^E^. Details have been described in the study by Menger et al. (2021),^19^ and a brief summary of the instrumental setup and settings is included in Supporting Information-A.

### Selection of TPs

A suspect list was created based on possible TPs of compounds included in the Swedish monitoring program for chemical pesticides in surface waters.^10^ This allowed for the creation of a suspect list consisting of suspects with a high likelihood of being present at the sites, assuming (partial) transformation of applied pesticides. The suspects were selected based on TPs listed in the Pesticide Properties Database (PPDB)^22^ of every target compound included in the monitoring program (*n* = 142, year 2017). Only “key metabolites” from the PPDB were selected (*n* = 214), that is, TPs identified by the PPDB in regulatory documents for pesticide authorization from, for example, the European Union, US EPA, or Health Canada.^22^

A number of parent compounds (*n* = 16), which were not part of the monitoring program in the year of the study, were additionally added to the suspect list because of their high interest from a monitoring perspective. These comprised (i) three compounds newly registered in Sweden, (ii) eight compounds used as a seed treatment, six of which were only registered for use in other EU countries, but potentially legally imported into Sweden in treated seeds, (iii) three active substances, for which only their TP (one for each) was included in the regular monitoring, and (iv) chlorothalonil, a fungicide that has been linked to adverse effects in bumblebee communities (e.g., McArt et al., 2017)^23^ and that is registered in other EU countries. All key metabolites (as indicated by the PPDB) of these 16 additional compounds (*n* = 28) were also added to the suspect list. Moreover, all 142 target analytes in the regular monitoring program (2017) were added to the suspect list. In summary, 400 compounds were included in the suspect list. The full list of compounds is given in Table SI-B1 in the Supporting Information and is also available online on the NORMAN Suspect List Exchange (list S78),^24^ on PubChem,^25^ and as a Zenodo data set.^26^

### Suspect Identification Workflow

The data treatment, which eventually led to the tentative identification of suspected compounds, was performed in a two-step approach. It combined the use of evidence from the HRMS instrument analysis with in-depth knowledge on pesticide properties and behavior, their TPs, and the characteristics of the sampling sites. The treatment of HRMS data was performed according to established suspect screening evaluation strategies described elsewhere, for example, ref (21), and is given in detail in Supporting Information-A. In brief, HRMS data were first preprocessed, and then, feasibility of suspect screening hits was evaluated based on the peak shape, retention plausibility, and in-depth scrutiny of fragmentation information using European Massbank, MetFrag,^27−29^ and manual checks. Feasibility of suspect screening hits was further studied considering meta data from the monitoring program including information on the application sites of the parent pesticides, known (historical) occurrence and behavior of the parent pesticides, degradation pathways and interpretation of persistence (PPDB), and levels of the pesticides and their TPs from other research and monitoring groups (based on the literature). Candidates with reasonable proofs considering both monitoring knowledge and experimental data were considered tentatively identified, and reference standards were purchased for final confirmation when commercially available.

### Semiquantification

Concentrations of newly confirmed compounds were estimated by means of semiquantitative analysis. Stored water from both sampling sites (E and M) was pooled separately, spiked with native solution of all newly confirmed compounds, and was extracted with the same SPE setup as used for field TIMFIE sampling to produce matrix-matched calibration curves with concentration levels of 0.1, 1, and 10 μg L^–1^. Additionally, a calibration curve without a matrix was prepared in methanol at concentration levels of 0.01, 0.1, 1, and 10 μg L^–1^. Blanks, both nonspiked pooled water and methanol, were included for every calibration curve.

Concentrations of newly confirmed TPs were estimated by comparing IS-normalized areas in the samples with IS-normalized areas in the matrix-matched calibration curve of the respective sampling site. Imazalil-*d*_5_ and clothianidin-*d*_3_ were used as IS in the positive ionization (PI) mode and negative ionization (NI) mode, respectively. Concentrations were reported as concentration ranges reflecting the uncertainty in quantification without prior method validation and limited access to relevant ISs. Four possible ranges were defined, viz., <0.1, 0.1–1, 1–10, and >10 μg L^–1^. In case of no linearity in the matrix-matched calibration curve, the TP was only reported as “detected”, and in case the lowest calibration point was not detected, the lowest concentration range was adjusted to <1 μg L^–1^.

## Results and Discussion

### Performance of the Screening Study Compared to Monitoring Data Based on Target Analytes

In total, 43 (site E) and 55 (site M) pesticides out of the 142 target compounds included in the regular monitoring were detected during the whole year of 2017.^10^ During the specific weeks in which the present suspect screening study was performed, the regular monitoring reported 36 (E) and 47 (M) compounds (∼85% of the total). This highlights the fact that our sampling covered periods with high pesticide presence in the streams. Therefore, high TP presence could be expected too. The results in this study for the TIMFIE samples analyzed using HRMS confirmed the occurrence of 27 (E) and 34 (M) of these target compounds (∼75% of the monitoring results), which showed that our screening method had good agreement with the results of the accredited methods used for monitoring. Target compounds were tracked in our screening study using standard solutions from the monitoring program, and thus, false positives were systematically avoided. However, it should be mentioned that the detection frequency of some pesticides was lower in our screening results compared to the monitoring results, which can be expected considering the low detection limits of the accredited target methods.^10,30^ This highlights the need for sensitive analytical methods and that environmental occurrence of pesticide TPs determined in our suspect screening study could be underestimated. Furthermore, it is likely that few target pesticides were systemically missed because they required different analytical methods (e.g., glyphosate). As the TIMFIE device is not dependent on power supply or batteries, and because of its low cost, small format, and ease of use, it is an interesting sampling technique to be considered also for other (suspect) screening studies. Also, because TIMFIE is a quantitative sampling technique, it gives the possibilities to perform semiquantification in these types of studies.

The transport of pesticides, as well as their TPs, to the aquatic environment is likely to be highly episodic, as it is affected by, for example, pesticide application, crop status, soil type, and weather conditions.^31^ Thus, to enable adequate monitoring and screening for this type of compounds, continuous sampling techniques, like the newly developed TIMFIE sampler, are advantageous compared to grab sampling.^19^ Despite the restrictions in sample volume given by the TIMFIE sampler, and consequently, relatively low preconcentration factors of approximately 200, we show in this study that sampling by TIMFIE can be an affordable and attractive choice for collecting representative samples in future suspect screening investigations, especially when (semi-) quantification is desired.

### Suspect Screening of Pesticide TPs

A total of 238 suspect screening hits were obtained across all samples after applying the suspect list (*n* = 258, excluding target compounds) to the preprocessed data sets (92 in PI and 146 in NI). After the peak shape and RT checks, 172 suspect screening hits remained (91 in PI and 81 in NI). Fifty-eight structures were discarded as false positives after in-depth investigation of the fragment spectra, and consequently, 114 suspect screening hits remained (53 in PI and 61 in NI). Monitoring knowledge was considered for these 114 structures (see section below), and finally, 42 candidates were considered tentatively identified based on chemical evidence and monitoring knowledge. Four suspects were detected in both, PI and NI, and thus, 38 unique structures were tentatively identified. This list would have been considerably shorter (*n* = 23) if it was based only on chemical evidences (viz., exact mass, fragmentation pattern, and RT plausibility), as most standard suspect screening workflows proceed,^32−34^ meaning that 19 additional candidates were considered tentatively identified thanks to the consideration of monitoring data. For example, reference spectra from European Massbank were available for only 6 of the 42 tentatively identified compounds, highlighting the lack of MS reference spectra for TPs. Several compounds had no entries in major chemical databases (e.g., PubChem or Chemspider), making it difficult to use in silico tools (like MetFrag) to increase the confidence in the identification. Thus, the application of monitoring knowledge as an additional source of relevant information was crucial to filter more compounds of interest. TPs with no clear chemical evidence toward their identification (e.g., no fitting fragments at low signal intensity) were kept if monitoring data showed the presence or use of their corresponding parent compounds at the respective sampling site. While compounds with a low confidence level would be discarded by following standard approaches, monitoring knowledge provided additional evidence to support their presence in our study allowing for the (tentative) identification of a higher number of potentially hazardous pesticide TPs. In this manner, 19 additional compounds that otherwise would have been discarded were further explored. Twenty-one pesticide TPs were retrospectively introduced to PubChem based on our study^25^ and are now available for future screening studies.

Reference standards could be purchased for 26 of the 38 unique candidates, and 11 pesticide TPs could subsequently be confirmed (azoxystrobin TP1, diuron TP1, folpet TP2, metazachlor TP2, atrazine TP3, chloridazon TP1, chloridazon TP2, fenpropimorph TP2, metalaxyl TP1, phenmedipham TP3, and thiacloprid TP1) (Table 1). Some of these newly confirmed TPs (e.g., chloridazon TP1 and TP2, atrazine TP3, metazachlor TP2, and azoxystrobin TP1) have already been reported in several other studies,^6,14,35−37^ while other TPs have not gained attention as environmental pollutants previously (e.g., fenpropimorph TP2, phenmedipham TP3, and thiacloprid TP1). Metalaxyl TP1 was recently tentatively identified in wastewater.^9^ Fifteen compounds were false positives and were not further elucidated (Table S2 in Supporting Information-A). One limitation in the identification of TPs was the lack of commercial reference standards for several chemicals (*n* = 12 out of 38 in our study), which hampered the confirmation of their identity. These compounds were considered tentatively identified with varying levels of confidence (Table 1 and are given in detail in Supporting Information-B3).^38^

**Table 1:**
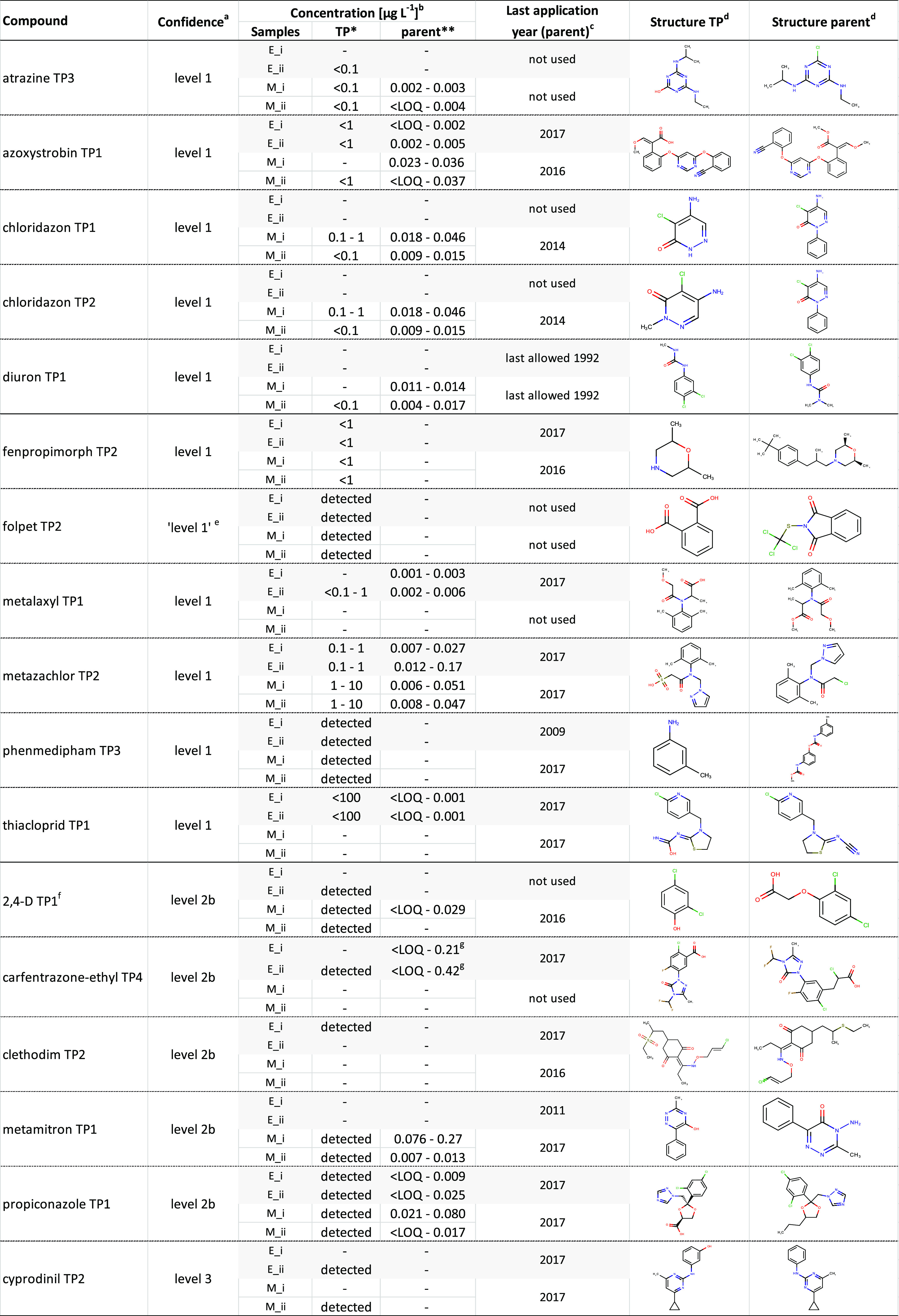

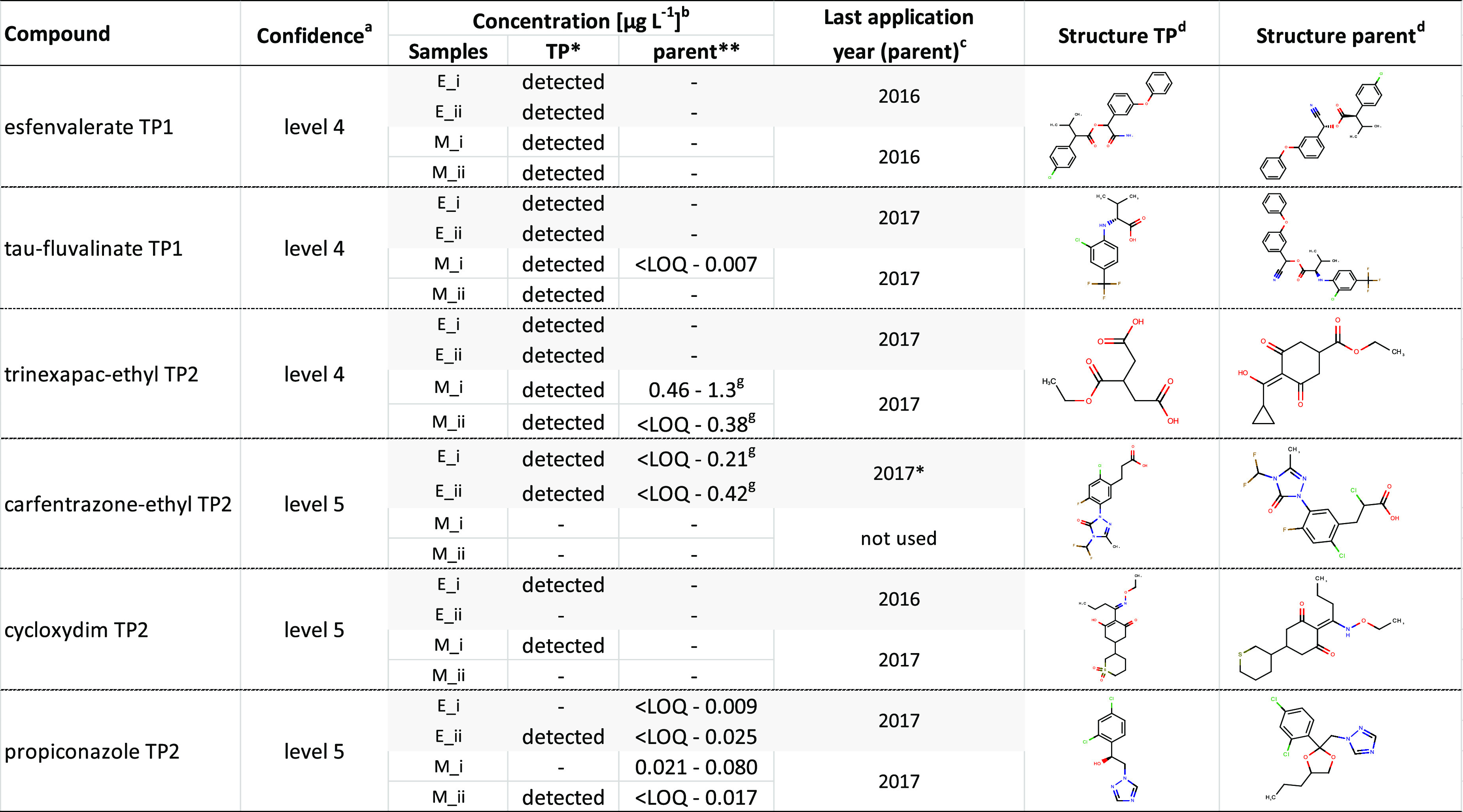
Newly (Tentatively) Identified Compounds

a Confidence level according to the study by Schymanski et al. (2014).

b Concentrations of the TP and the corresponding parent compound at sampling sites E and M during sampling campaigns 1 and 2 (depicted as “_i” and “_ii”, respectively). Concentration values were determined as concentration ranges in our suspect screening study through semiquantification (*) or extracted from data from the Swedish national monitoring for pesticides, which uses accredited methods (**) (Boye et al., 2019). Periods in which compounds were not detected were marked “-”.

c Last year of application of the parent pesticide per sampling site and the structures of the TP and the corresponding parent pesticide.

d JChem for Excel was used for displaying the chemical structures of the TP and the respective parent pesticide (JChem for Office, 20.14.0.668, ChemAxon, https://www.chemaxon.com).

e Nondistinguishable isomer theoretically possible despite matching reference standard information.

f Structure can be transformed from other known parent pesticides.

g Concentration values from a different TP, which was included in the monitoring program.

Table 1 shows the suspect screening findings including the confidence level, concentration (or presence), structure of both the TP and the parent pesticide, and last application year of the parent pesticides (for more details, see Supporting Information-B3). Occurrence of TPs generally followed the occurrence of the respective parent compounds, which in turn in many cases also had registered uses at the site(s) of detection during the year of the study (2017). However, several newly (tentatively) identified TPs were also detected years after the parent compounds had been used the last time, for example, diuron TP1, chloridazon TP1, and chloridazon TP2. This confirms the relevance of concerns for stable pesticide TPs occurring in the environment under natural conditions.^12^ However, it also happened that some TPs were detected at sites where the corresponding parent compounds were not present (e.g., folpet TP2, fenpropimorph TP2, and phenmedipham TP3). A reason for this could be higher persistence of the TP or fast transformation rate of the parent compound, which is known for some compounds and is the reason why some TPs, for example, trinexapac and prothioconazole-desthio, are already included in the regular monitoring in Sweden. Another reason could be different mobilities of the TP compared to its parent compound. TPs generally are more polar than their parent compounds,^5,39^ which make them more mobile and less retained in the soil, and therefore reach water bodies more easily. A physicochemical property commonly used for predicting mobility of pesticides in the environment is the soil organic carbon to the water partitioning coefficient (*K*_foc_).^40^ For 16 of the 23 newly (tentatively) identified TPs, a *K*_foc_ value was available in the PPDB, whereof 12 had a lower *K*_foc_ (higher mobility) than the respective parent pesticide (Table SI-B2).

Concentration ranges could be determined for four newly confirmed pesticide TPs (viz., chloridazon TP1, chloridazon TP2, metazachlor TP2, and metalaxyl TP1), which allowed for comparison between concentration levels of parent compounds and TPs. For five confirmed TPs, only upper concentration limits could be determined and the concentrations for two confirmed TPs could not be estimated, as no linearity of the calibration curve could be achieved. All four TPs for which a concentration range could be determined were present in comparable or higher concentrations than their respective parent compounds (Table 1).

The results demonstrated that considering prior monitoring knowledge instead of, for example, only gathering information on structurally predicted TPs or general literature information is a powerful tool in the selection of relevant pesticide TPs. Eleven compounds could be unequivocally identified (level 1), and five compounds were tentatively identified at a high confidence level (level 2b), from a custom-built list of 258 suspects, which are not regularly investigated. This confirmed the value of using monitoring knowledge to craft a highly relevant suspect list adjusted for specific sampling sites in the environment, which in return created possibilities for in-depth investigations even at low signal intensities.

It should be mentioned that several compounds were tentatively identified at low confidence levels 4 and 5 (*n* = 6). In these cases, clear uncertainties about the true identities remain because of the lack of chemical evidence supporting their tentative identities. However, monitoring data supported the presence of these compounds in the detected samples, which is why they are reported here and a reason for consideration in further studies.

### Benefit of Integrating Monitoring Data into Suspect Screening

**Figure 1:**
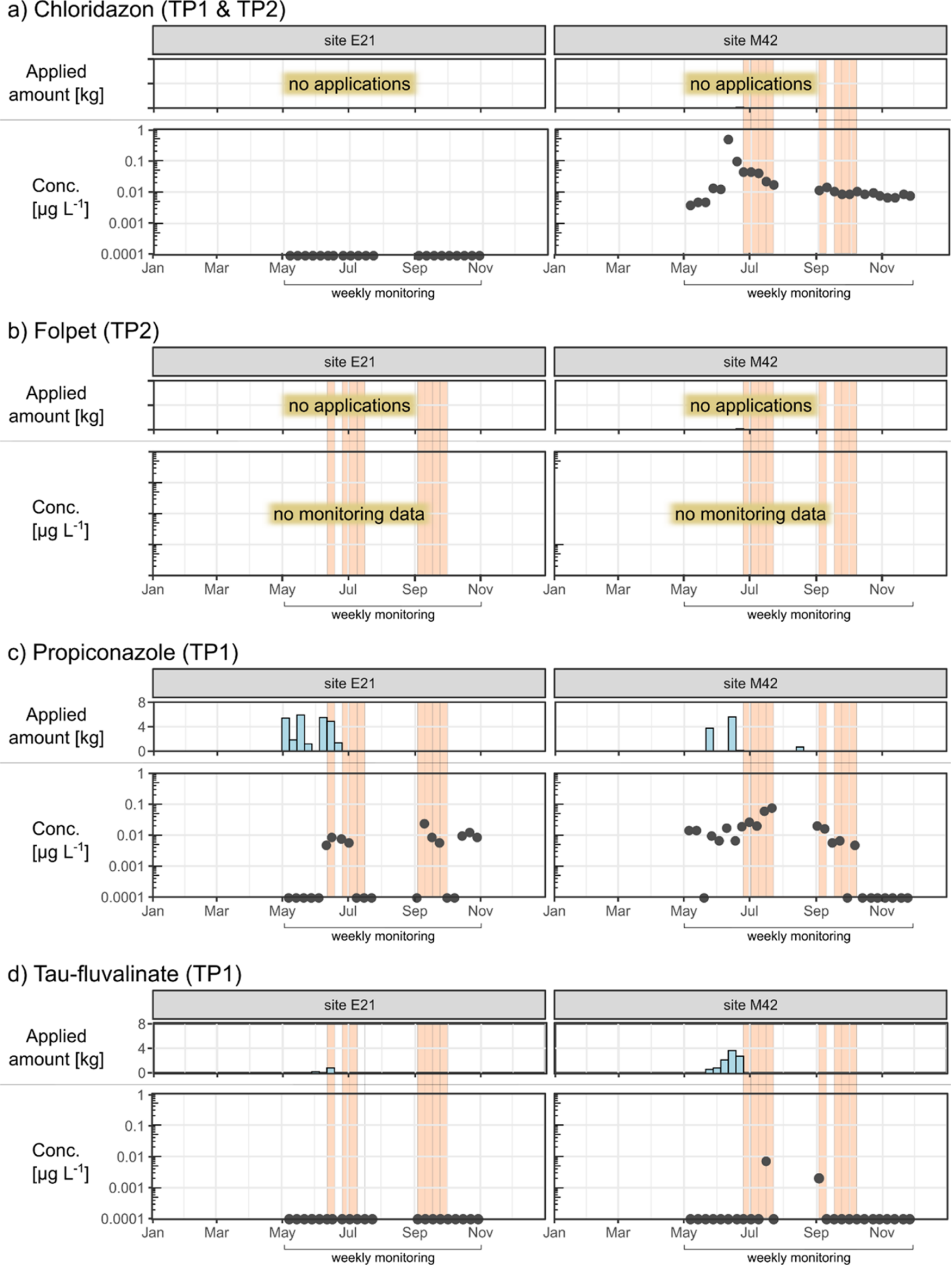
Occurrence of selected (tentatively) identified pesticide TPs (orange bars) in context to monitoring data from 2017, namely, the total amount of the applied pesticide per week (“applied amount”) and average weekly concentration (concn) of the respective parent compounds.

Four examples of newly (tentatively) identified suspects were selected to showcase and discuss the role and benefit monitoring data played in our newly developed suspect screening approach (Figure 1).

#### Chloridazon

Two chloridazon TPs (TP1 and TP2, i.e., desphenyl-chloridazon and methyl-desphenyl-chloridazon, respectively) produced suspect screening hits across all samples from site M and could quickly be assigned a confidence level 2b,^38^ as diagnostic fragments listed in European Massbank matched experimental data [viz., 100.99010 and 116.99759 *m*/*z* (chloridazon TP1) and 87.99492, 116.99759, and 130.00550 *m*/*z* (chloridazon TP2)]. The main use of chloridazon in Sweden has been on sugar beets (only grown at site M), and chloridazon has regularly been detected at site M, for example, during the year of our study (2017) (Figure 1a), although no application occurred during that year.^10^ Reference standards for these TPs were commercially available, which led to the confirmation of both TPs (level 1). Both TPs could be semiquantified, and chloridazon TP1 and TP2 were both detected at concentration ranges close to or higher than the concentration levels of the parent pesticide (Table 1). This highlighted the concerns regarding these TPs, especially considering that they were detected years after chloridazon was last applied in this area (May 2014). Chloridazon TP1 and chloridazon TP2 are known environmental contaminants that have been detected in, for example, ground, surface, and drinking water.^11,37,41^ Chloridazon TP1 is resistant to degradation,^11^ and both TPs have been detected at concentration levels above the parent pesticide levels (ground water) and frequently exceeded health-based parametric values.^41,42^ Therefore, our study confirms existing concerns regarding desphenyl-chloridazon and methyl-desphenyl-chloridazon as environmental contaminants, specifically in surface water. Chloridazon and its TPs showcased the optimal scenario for a suspect screening with integrated monitoring knowledge. Established HRMS data evaluation methodologies led to quick and confident tentative identifications of the suspects, which were fully supported by monitoring data, and reference standards were readily available for immediate unequivocal confirmation and semiquantification.

#### Folpet

Folpet TP2 (phthalic acid) was, like chloridazon TP1 and TP2, quickly and confidently tentatively identified with a matching main fragment from European Massbank (viz., 121.0295 *m*/*z*) and high score in MetFrag (level 2a). However, from a monitoring perspective, these findings could not be supported. The parent compound (folpet) had just been reregistered for use in Sweden and had no uses registered at either sampling site according to the monitoring database (Figure 1b). A reference standard was purchased, and folpet TP2 was confirmed in most samples. While the identity of the compound was confidently determined as phthalic acid (unequivocal confirmation was not possible due to indistinguishable para-/meta-isomers), its origin in our study most certainly cannot be explained by the transformation of folpet into its TP. Instead, phthalic acid could have originated from transformation from other parent compounds (e.g., hydrolysis of phthalic anhydride or phthalate esters) or as a byproduct from chemical industry (e.g., manufacture of phthalic anhydride).^43,44^ Furthermore, phthalic acid has been included in other suspect screening studies, for example, in a study aimed at pesticide TPs in which the compound was listed as a potential TP of other pesticides, besides folpet, for example, napropamide, phosmet, and acequinocyl.^5^ This indicates that phthalic acid can have many different origins, which supports our hypothesis that “folpet TP2” likely originated from a different source than the transformation of folpet in our study. The case of phthalic acid highlights the importance of putting findings of a suspect screening into the correct context to avoid false conclusions. Under different circumstances, the identified structure could have been falsely reported as a TP of the arguably wrong parent compound, which can be crucial when control measures are designed for newly identified compounds of concern.

#### Propiconazole

Little confidence was initially available for propiconazole TP1, as the structure was not listed in PubChem, which reduced the power of MetFrag, and no reference spectra were listed in European MassBank. Only the presence of two chlorines in the structure could be confirmed. However, the confidence in the tentative identification greatly increased, once knowledge from the monitoring program was additionally considered. Two diagnostic fragments common to the suspected TP and its parent pesticide were confirmed in PI, viz., 87.04406 and 186.97120 *m*/*z*. Furthermore, the presence of propiconazole TP1 at both sampling sites was supported by the fact that propiconazole had registered uses at both sites during 2017 and had been detected regularly throughout the monitoring year (Figure 1c). There was no information publicly available about this compound, which highlighted the uncertainty regarding, for example, environmental occurrence, toxicity, and, ultimately, risks of this compound. The case of propiconazole TP1 showed how confidence in a tentative identification can greatly be increased when experimental findings can be supported by monitoring knowledge. It also, however, highlighted the limitations when no reference material is available. Unequivocal structure confirmation remains impossible, and without reference standards, new findings cannot be implemented into target methods and reliable quantification cannot be performed. This leaves a knowledge gap regarding the environmental risks of these newly tentatively identified compounds, which will be hard to overcome.

#### Tau-fluvalinate

Tau-fluvalinate TP1 (anilino acid) was tentatively identified in most samples (Figure 1d) at confidence level 4, with several possible alternatives from MetFrag and no available reference spectra in European Massbank, but a clear isotope pattern matching one chlorine. Even after detailed investigation, no diagnostic fragments could be determined (remained at level 4), which would likely lead to discarding in most other suspect screening studies. In our study, however, monitoring knowledge and in-depth knowledge about pesticide properties led to high interest in this compound. The parent compound, tau-fluvalinate, a highly potent pyrethroid insecticide, had been used at both sampling sites (higher quantity used at site M than at site E) and had been detected only twice at site M at low concentrations (<0.01 μg L^–1^) during all of 2017 (Figure 1d). Tau-fluvalinate is likely to sorb to soil particles (*K*_foc_ = 186,000 mL g^–1^) (Table SI-B2), which makes it unlikely to leach to surface water and, therefore, explains the rare detection in surface water during monitoring. Conversely, tau-fluvalinate TP1 has a much lower *K*_foc_ at 242 mL g^–1^, making it more mobile and, therefore, more prone to leaching to surface water, which could explain its frequent detection compared to its rarely detected parent compound. While the frequent occurrence of tau-fluvalinate TP1 was supported by monitoring knowledge, its low signal intensities consequently led to poor fragment information and the absence of a commercially available reference standard resulted in an arguably low confidence in its structure (level 4). This, for once, laid bare the limitations of screening for trace contaminants close to instrumental detection limits and the importance of analytical reference standards but also highlighted how implementation of meta data (e.g., monitoring knowledge) can be crucial to filter compounds of concern, especially when instrumental and/or software limitations would result in too low confidence and likely discarding.

These four examples highlight the advantages of integrating monitoring knowledge into suspect screening evaluation. Similar concepts of utilizing meta data (e.g., monitoring data) to enhance the prioritization and identification of potentially underinvestigated chemicals have been developed before, for example, using chemical market data to prioritize micropollutants in surface water impacted by wastewater treatment facilities or pesticides and pesticide TPs in groundwater.^5,18,21^ Another example is the consideration of reference and patent information for scoring in in silico fragmentation software.^29^ However, even when considering meta data in the workflows of HRMS-based screening studies, the prioritization and elucidation of compounds with very little to no available reference information (e.g., reference standards, reference spectra, or even only entries in chemical compound databases) stays challenging, relies on time-intensive investigations of the fragment spectra, and therefore, remains unattractive.

### Benefits for Monitoring Programs to Engage with Suspect Screening

From the perspective of a monitoring program, a suspect screening can be a promising and reliable strategy to investigate whether current analysis methods cover the most relevant compounds present in the environment. In our study, we confirmed the occurrence of 11 pesticide TPs (plus 12 tentatively identified TPs), which highlighted that the current monitoring was missing compounds of potential concern. It is, however, important to carefully evaluate the environmental relevance of newly identified compounds to assess whether they should be added to a monitoring program, as it is of paramount importance to ensure a cost-effective monitoring. Some of the newly identified TPs were semiquantified during our study and exceeded the concentrations of the respective parent compounds (e.g., chloridazon TP1 and metazachlor TP2), and thus, they can be considered environmentally relevant. This shows how a suspect screening with integrated semiquantification can provide highly relevant information to a monitoring program, which allows for direct interpretation and possible implementation of findings within a comparatively short timeframe.

For the 11 newly confirmed pesticide TPs (level 1), inclusion in regular monitoring is technically a straightforward process because reference standards are already readily available. However, implementing the findings from a suspect screening still poses challenges in cases where a reference standard is not commercially available. In this study, a high confidence (level 2b) was reached for several compounds, but lack of available reference standards makes it currently not possible to include them in any target method used in monitoring. For some cases, where a finding is especially interesting, it might, however, be viable to have the reference standard synthesized by a manufacturer. One such example from this study is tau-fluvalinate TP1 (anilino acid), which was identified at a confidence level 4, but an unequivocal identification of the compound could benefit the monitoring program. The parent pesticide tau-fluvalinate is a pyrethroid insecticide, which is highly toxic to fish and aquatic invertebrates (e.g., a chronic 21 day no observed effect concentration (NOEC) for *Daphnia magna* at 0.02 μg L^–1^).^45^ In such cases, where the parent pesticides are known to be hazardous (e.g., for their very high toxicity), concerns regarding TPs whose presence was suggested in the environment arise, especially when the TPs display close structural similarity to the parent compounds. While the risk for aquatic organisms from anilino acid has already been assessed (something which cannot be expected for most TPs) and was assessed as low risk,^45^ confirmation and quantification of this TP could still be of interest. Environmental concentration levels of anilino acid remain unknown and are needed for, for example, risk assessment, mass load calculations and, ultimately, a better understanding of the fate of tau-fluvalinate in the environment.

Developing suspect screening studies based on monitoring interests has the advantage that findings can be implemented faster into monitoring programs, which becomes highly relevant in a regulatory context. Environmental contamination with pesticides and (unknown) pesticide TPs is of lasting concern and was supported because HRMS-based screening studies have started exploring the space of unknown environmental contaminants and highlighted new potential threats, for example, with regards to persistent and mobile organic compounds (PMOCs).^46^ Polar TPs, like the polar pesticide TPs tentatively identified in our study, have been highlighted specifically as one major contributing fraction to unknown PMOCs,^46^ and with our study, we managed to address this knowledge gap. However, the lack of available reference standards once again highlighted the limits of current identification approaches, which translates directly into the inability to monitor these compounds.

## Supporting Information Available

Information on chemicals used during extraction and analysis, instrumental setup and settings of the UPLC-QTOF system, data treatment during suspect screening including parameters and cut-off values, plots of precipitation, discharge and number of pesticide applications at the two study sites during 2017, list of reference standards purchased for confirmation, and list of false positive compounds (PDF)
Complete suspect list, *K*_foc_ values from PPDB of (tentatively) identified compounds, and detailed result summary table containing all (tentatively) identified pesticide TPs and their respective parent compounds (XLSX)

## Notes

Complete suspect list online at NORMAN Suspect List Exchange (list S78) (https://www.norman-network.com/nds/SLE/) and on PubChem (https://pubchem.ncbi.nlm.nih.gov/classification/#hid=101), and as a Zenodo data repository (https://zenodo.org/record/4687925).
