# Supplementary material for: Identification of Pesticide Transformation Products in Surface Water Using Suspect Screening Combined with National Monitoring Data
Source: Environ Sci Technol. 2021 Jul 22;55(15):10343–53. doi: 10.1021/acs.est.1c00466 (PMC8383268; doi:10.1021/acs.est.1c00466)
Supplement: Supplementary file 1 — es1c00466_si_001.pdf [file es1c00466_si_001.pdf]

Supporting Information

Identification of pesticide transformation products in surface water using suspect screening combined with national monitoring data

Frank Menger[1,*], Gustaf Boström[1], Ove Jonsson[1], Lutz Ahrens[1], Karin Wiberg[1], Jenny Kreuger[1] and Pablo Gago-Ferrero[2,3]

[1]Department of Aquatic Sciences and Assessment, MVM building, Swedish University of Agricultural Sciences (SLU), SE-75007 Uppsala, Sweden

[2]Catalan Institute for Water Research (ICRA), Carrer Emili Grahit 101, 17003 Girona, Spain

[3]Universitat de Girona, Girona, Spain

[*]Corresponding author: Frank Menger

Tel: +46 72 289 46 51

E-mail: Frank.menger@slu.se

Affiliation address: Bo 7050, SE-750 07 Uppsala, Sweden

This Supporting Information A (SI-A) contains 5 pages, 3 texts, 1 figure and 2 tables.

Contents

Chemicals used during extraction and analysis S2
Instrumental setup and settings S2
HRMS-data treatment during suspect screening........ S3
Figures........ S4
Tables S5
References S6

List of figures and tables

Figures

Figure S1 Precipitation, discharge, pesticide applications and sampling occasions S4

Tables

Table S1 List of purchased standards S5
Table S2 List of false positives S6

Chemicals used during extraction and analysis

Methanol, acetonitrile, isopropyl alcohol of HPLC grade, acetic acid (99.8%), ammonia (4 mol L^{-1} in methanol), and ammonium acetate of p.a. grade were purchased from Sigma-Aldrich (Steinheim, Germany), 25% ammonia solution and ammonium formate from Sigma-Aldrich (Sweden), and acetone of pesticide residue analysis grade and formic acid (99−100%) were purchased from VWR (Fontenay-sous-Bois, France). Water was purified using a Milli-Q Advantage A10 system from Merck Millipore (Darmstadt, Germany).

Instrumental setup and settings

Separate injections were performed for positive and negative ionization mode (PI and NI, respectively). Aliquots of 10 µL were injected on an Acquity HSS T3 column (100 mm × 2.1 mm, 1.8 µm) in PI mode and on an Acquity BEH C18 column (50 mm × 2.1 mm, 1.7 µm) in NI mode. Guard columns of the respective packing material were used. Ultrapure water with 5 mM ammonium formate buffer and 0.01% formic acid (PI) and with 5 mM ammonium acetate buffer with 0.01% ammonia (NI) was used as aqueous phase and the organic phase consisted of acetonitrile with 0.01% formic acid (PI) or with 0.01% ammonia (NI). The chromatographic run time was 21 min in both modes and a flow of 0.5 mL min^{-1} was applied. The initial conditions of the elution gradient were 5% organic phase and were kept at the start of a run for 0.5 min before increasing to 95% by 16 min. The organic content was then further increased to 99% within 0.1 min and kept for 3 min before starting conditions were restored and held for 2 min. Data was acquired in MS^E, which is a data independent acquisition mode alternating between low collision energy (CE) and high CE, and the mass range of the QTOF was set to 50 m/z to 800 m/z with a scan time of 0.25 s. The low CE channel was set to 4 eV and the high CE was a ramp from 10 eV to 45 eV. The mass-axis was calibrated daily from m/z 50 to 1200 with a 0.5 M sodium formate solution in 2-propanol/water (90:10). The resolution was ~30 000 at 556.28 *m/z* with leucine enkephalin used as lock spray (for lock mass correction).

HRMS-data treatment during suspect screening

Data pre-processing, *i.e.* basic reduction of raw data complexity, was performed using UNIFI (Version 1.8.0.0) and included intensity thresholds (250 counts for PI and 100 counts for NI in low CE, and 50 counts for both PI and NI in high CE) and componentization, *i.e.* grouping of signals belonging to the same molecular structure. Suspect screening hits were obtained using a mass error threshold of ≤2.5 mDa for the mass difference between the protonated (PI) or de-protonated (NI) suspect and the measured exact mass of the components. The number of suspect screening hits was reduced by manually checking for and discarding noise-peaks based on S/N ratio (≥3) and peak shape. Features with clearly unfeasible chromatographic retention times (RTs), as compared to the respective known parent pesticide RTs, were discarded as well. Suspect screening hits were investigated towards tentative identification through in-depth scrutiny of the high CE spectra. If available, experimental fragment spectra were compared to reference spectra from the spectral library European MassBank (Horai et al., 2010), and the suspected structures were weighted against other possible compounds that could have explained the experimental fragment data by using the in silico fragmentation software MetFrag (Ruttkies et al., 2019, 2016). MetFrag was operated using the statistical software R (version 3.6.0) via the R package MetFragR. PubChem was chosen as compound database and fragment score, number of patents and number of references were considered as scoring terms. Observable characteristic isotopic patterns were considered additional evidence for suspected halogenated molecular formulas.

Figures

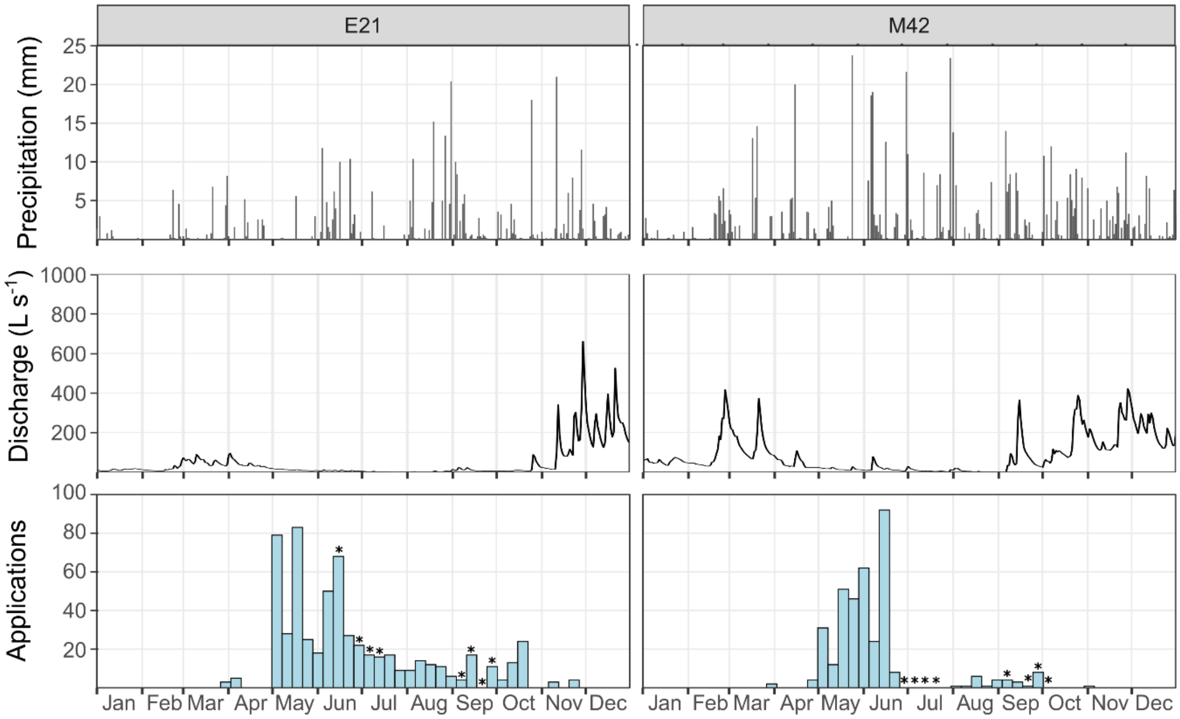

Figure S1 Precipitation, discharge, and number of applications per week plotted separately for catchments E21 and M42 during the year 2017. Sampling occasions are indicated by asterixis.

Tables

Table S1 Tentatively identified compounds that were bought for final confirmation.

**Compound**	**SMILES**	**vendor**
2,4-D TP3	Oc1ccc(Cl)cc1	Sigma-Aldrich
alachlor TP1	CCc1cccc(c1N(COC)C(=O)CS(=O)(=O)O)CC	Sigma-Aldrich
alachlor TP2	CCc1cccc(c1N(COC)C(=O)C(=O)O)CC	Sigma-Aldrich
atrazine TP3	c1(nc(nc(n1)O)NCC)NC(C)C	Sigma-Aldrich
azoxystrobin TP1	CO/C=C(\c1ccccc1Oc2cc(ncn2)Oc3ccccc3C#N)/C(=O)O	LGC-standards
chloridazon TP1	NC=1/C=N\NC(=O)C=1Cl	abcr
chloridazon TP2	Cl\C1=C(\C=N/N(C1=O)C)N	LGC-standards
diuron TP1	c1(cc(c(Cl)cc1)Cl)NC(NC)=O	Sigma-Aldrich
fenitrothion TP	c1(c(cc(O)cc1)C)[N+](=O)[O-]	abcr
fenpropimorph TP2	O1C(C)CNCC1C	Sigma-Aldrich
flupyrsulfuron-methyl TP3	COc1cc(OC)nc(N)n1	Sigma-Aldrich
folpet TP2	O=C(O)c1ccccc1C(=O)O	Sigma-Aldrich
imazalil TP1	O=C(c1ccc(Cl)cc1Cl)Cn2ccnc2	abcr
MCPA TP1	c1(c(ccc(c1)Cl)O)C	Sigma-Aldrich
metalaxyl TP1	CC(N(C(=O)COC)c1c(C)cccc1C)C(=O)O	Sigma-Aldrich
metazachlor TP2	Cc1cccc(c1N(Cn2cccn2)C(=O)CS(=O)(=O)O)C	LGC-standards
metribuzin TP1	O=C/1NC(=O)N\N=C\1C(C)(C)C	LGC-standards
metribuzin TP2	O=C/1NC(=O)N\N=C\1C(C)(C)C	LGC-standards
metsulfuron-methyl TP2	O=S(N)(=O)c1ccccc1C(=O)O	abcr
phenmedipham TP2	Oc1cccc(N)c1	Sigma-Aldrich
phenmedipham TP3	c1c(cccc1N)C	Sigma-Aldrich
picoxystrobin TP2	c1cc(nc(c1)O)C(F)(F)F	abcr
pirimicarb TP2	Cc1c([nH]c(nc1=O)NC)C	LGC-standards
pyrethroid TP	c1ccc(cc1)Oc2cccc(c2)C(=O)O	Sigma-Aldrich
thiacloprid TP1	c1cc(ncc1CN\2CCS/C2=N\C(=N)O)Cl	Sigma-Aldrich
triadimenol	Clc2ccc(OC(n1ncnc1)C(O)C(C)(C)C)cc2	Sigma-Aldrich

Table S2 List of false positive compounds.

Compound	PubChem CID	Formula
2,4-D TP3	4684	C_6H_5OCl
alachlor TP1	115236	$C_{14}H_{21}NO_5S$
alachlor TP2	14924483	$C_{14}H_{19}NO_4$
fenitrothion TP	17412	$C_7H_7NO_3$
flupyrsulfuron-methyl TP3	118946	$C_6H_9N_3O_2$
imazalil TP1	32232	$C_{11}H_8Cl_2N_2O$
MCPA TP1	14855	C_7H_7ClO
metribuzin TP1	41909	$C_7H_{12}N_4O_2$
metribuzin TP2	40299	$C_7H_{11}N_3O_2$
metsulfuron-methyl TP2	69436	$C_7H_7NO_4S$
phenmedipham TP2	11568	C_6H_7NO
picoxystrobin TP2	10154195	$C_6H_4F_3NO$
pirimicarb TP2	135401759	$C_7H_{11}N_3O$
pyrethroid TP	19539	$C_{13}H_{10}O_3$
triadimenol	41368	$C_{14}H_{18}ClN_3O_2$

References

Horai, H., Arita, M., Kanaya, S., Nihei, Y., Ikeda, T., Suwa, K., Ojima, Y., Tanaka, Kenichi, Tanaka, S., Aoshima, K., Oda, Y., Kakazu, Y., Kusano, M., Tohge, T., Matsuda, F., Sawada, Y., Hirai, M.Y., Nakanishi, H., Ikeda, K., Akimoto, N., Maoka, T., Takahashi, H., Ara, T., Sakurai, N., Suzuki, H., Shibata, D., Neumann, S., Iida, T., Tanaka, Ken, Funatsu, K., Matsuura, F., Soga, T., Taguchi, R., Saito, K., Nishioka, T., 2010. MassBank: a public repository for sharing mass spectral data for life sciences. J. Mass Spectrom. 45, 703–714. https://doi.org/10.1002/jms.1777

Ruttkies, C., Neumann, S., Posch, S., 2019. Improving MetFrag with statistical learning of fragment annotations. BMC Bioinformatics 20. https://doi.org/10.1186/s12859-019-2954-7

Ruttkies, C., Schymanski, E.L., Wolf, S., Hollender, J., Neumann, S., 2016. MetFrag relaunched: incorporating strategies beyond in silico fragmentation. J. Cheminformatics 8. https://doi.org/10.1186/s13321-016-0115-9

Schymanski, E.L., Jeon, J., Gulde, R., Fenner, K., Ruff, M., Singer, H.P., Hollender, J., 2014. Identifying Small Molecules via High Resolution Mass Spectrometry: Communicating Confidence. Environ. Sci. Technol. 48, 2097–2098. https://doi.org/10.1021/es5002105
